# *Isthmin 1* (*ism1*) is required for normal hematopoiesis in developing zebrafish

**DOI:** 10.1371/journal.pone.0196872

**Published:** 2018-05-14

**Authors:** Arturo Berrun, Elena Harris, David L. Stachura

**Affiliations:** 1 Department of Biological Sciences, California State University Chico, Chico, CA, United States of America; 2 Department of Computer Sciences, California State University Chico, Chico, CA, United States of America; University of Colorado Boulder, UNITED STATES

## Abstract

Hematopoiesis is an essential and highly regulated biological process that begins with hematopoietic stem cells (HSCs). In healthy organisms, HSCs are responsible for generating a multitude of mature blood cells every day, yet the molecular pathways that instruct HSCs to self-renew and differentiate into post-mitotic blood cells are not fully known. To understand these molecular pathways, we investigated novel genes expressed in hematopoietic-supportive cell lines from the zebrafish *(Danio rerio)*, a model system increasingly utilized to uncover molecular pathways important in the development of other vertebrate species. We performed RNA sequencing of the transcriptome of three stromal cell lines derived from different stages of embryonic and adult zebrafish and identified hundreds of highly expressed transcripts. For our studies, we focused on *isthmin 1* (*ism1)* due to its shared synteny with its human gene ortholog and because it is a secreted protein. To characterize *ism1*, we performed loss-of-function experiments to identify if mature blood cell production was disrupted. Myeloid and erythroid lineages were visualized and scored with transgenic zebrafish expressing lineage-specific markers. *ism1* knockdown led to reduced numbers of neutrophils, macrophages, and erythrocytes. Analysis of clonal methylcellulose assays from *ism1* morphants also showed a reduction in total hematopoietic stem and progenitor cells (HSPCs). Overall, we demonstrate that *ism1* is required for normal generation of HSPCs and their downstream progeny during zebrafish hematopoiesis. Further investigation into *ism1* and its importance in hematopoiesis may elucidate evolutionarily conserved processes in blood formation that can be further investigated for potential clinical utility.

## Introduction

Hematopoiesis is an essential cellular process in which hematopoietic stem cells (HSCs) differentiate into the multitude of different cell lineages that comprise mature blood[1–3]. HSCs must self-renew and persist for an organism’s lifespan to replenish the mature, post-mitotic blood cells that are constantly being recycled, ensuring that the system is never depleted[4, 5]. The control of this recycling and replacement of blood cells is regulated by an intricate set of signaling molecules and molecular pathways, many of which are still enigmatic. Improper regulation of hematopoiesis can result in serious diseases such as anemia, thrombocytopenia, neutropenia, and leukemia, so understanding these signaling pathways is of clinical relevance.

In vertebrates, HCSs first arise from hemogenic endothelium located in the floor of the dorsal aorta[6–13]. This occurs at embryonic day (E) 10.5 in mice[6], between E27-40 in humans[14], and between 36–52 hours post fertilization (hpf) in the developing zebrafish embryo[7, 8]. Lineage tracing studies in zebrafish[7] and mice[15, 16] indicate that the HSCs that arise during this time give rise to all hematopoietic cells for the organism’s lifespan. Importantly, studies in mice and humans indicate that HSCs don’t directly differentiate into mature blood cells. Instead, they differentiate into populations of restricted hematopoietic stem and progenitor cells (HSPCs); common lymphoid progenitors (CLPs)[17, 18], which eventually produce T, B, and natural killer (NK) cells, and common myeloid progenitors (CMPs)[19, 20] that eventually generate granulocytes, erythrocytes, macrophages, and platelets. Downstream of CMPs are megakaryocyte erythroid progenitors (MEPs) that generate erythrocytes and platelets, and granulocyte macrophage progenitors (GMPs) that generate basophils, eosinophils, neutrophils, and macrophages[19, 20]. Together these HSPCs help maintain the multitude of blood cells in healthy adult organisms.

HSPC differentiation is a developmentally restrictive process, controlled by a multitude of cytokines. These small, extracellular proteins influence HSPCs to self-renew and/or undergo stepwise differentiation into mature blood cell lineages and are secreted in hematopoietic niches, mainly by stromal cells that are found in hematopoietic-supportive tissues and organs (reviewed in [21–23]). These factors then bind to receptors on the surface of HSPCs to mediate a multitude of different downstream cellular responses. Identification and elucidation of the downstream molecular events activated by cytokines is of key interest due to their essential role in hematopoietic regulation; improper differentiation of HSPCs can lead to an accumulation of immature cells, causing the development of lymphoma and leukemia.

To study hematopoiesis and HSPC biology, many laboratories utilize *Danio rerio* (zebrafish), which have become a promising model system for many reasons (reviewed in [24, 25]). First, they are the phylogenetically lowest vertebrate model system that has a similar circulatory and hematopoietic system to humans, including adaptive immunity (reviewed in [26]). Secondly, zebrafish are transparent and develop *ex utero*; within 48 hpf functional HSPCs are present[7, 8, 27–30]. Due to the fact that a multitude of hematopoietic-specific fluorescent transgenic zebrafish lines currently exist (reviewed in [24, 31]), HSPCs can be visualized, isolated, and studied in early embryos, a feat not possible in mammals. The fact that zebrafish are fecund, generating hundreds of embryos per clutch, allows large sample sizes and experimental replication. Finally, because zebrafish develop quickly and externally, they are excellent for mutagenesis studies[32–36] and screening compounds that have utility for treating human diseases[37–41]. For all of these reasons, the zebrafish has become a powerful model for studying normal hematopoiesis and its dysregulation during disease.

HSPCs, the cells mutated during the onset of hematopoietic disease, are not easily grown with traditional cell culture techniques because they require specific microenvironments and cytokine signals to keep them proliferating and to encourage their differentiation into mature blood cells. Studies performed in mice[42–45] and humans[46, 47], isolating and growing HSPCs on stromal cell lines, were important for elucidating cytokines and other signaling molecules required for HSPC proliferation and differentiation. The recent generation of hematopoietic-supportive zebrafish stromal cell lines from sites of embryonic[48, 49] and adult[50] hematopoiesis now allows functional testing of HSPCs in zebrafish. Putative hematopoietic-supportive factors expressed in zebrafish cell lines have been identified with RNA sequencing (RNA-seq), comparing the transcriptome of these cells to that of non-hematopoietic supportive zebrafish stroma[48]. Additionally, comparison of their transcriptome to mammalian hematopoietic-supportive stroma allows investigation of transcripts conserved throughout vertebrate evolution[51].

One interesting transcript uniquely upregulated in all hematopoietic-supportive zebrafish cell lines was isthmin 1 (*ism1)*, a gene first identified in the midbrain–hindbrain boundary (MHB) of *Xenopus[52]. ism1* is expressed in lymphocytes,[53] bone marrow[54], and in embryonic blood islands[52], and encodes a secreted 60 kDa protein containing a copy of the thrombospondin repeat (TSR) region, a domain involved in cell migration and tissue remodeling[55]. Importantly, it is also expressed in mouse and chicken lateral plate mesoderm, tissue which gives rise to blood in the developing embryo[54]. *ism1* is also implicated in angiogenesis; addition of ISM1 protein into matrigel plugs with murine tumors resulted in decreased endothelial capillary networks and decreased overall tumor growth[55]. Additionally, *ism1* morphant zebrafish exhibit decreased inter-segmental vessels (ISVs)[55]. Importantly, *ism1* levels are increased in response to the upregulation of Wnt signaling[56], implicating its role in early embryonic processes such as cell fate specification, migration, and the beginning of definitive hematopoiesis. Finally, *ism1* is co-expressed with fibroblast growth factor ligands that are essential for HSC specification during this developmental period[52]. *ism1*’s high expression within hematopoietic-supportive stromal cell lines coupled with its expression during development in blood-forming tissues and co-expression with essential hematopoietic factors indicated that *ism1* was potentially involved in the formation and modulation of HSPCs.

To understand *ism1*’s role within developmental hematopoiesis, we performed loss-of-function experiments, which indicate that *ism1* morphants have reduced mature erythroid and myeloid cells. Additionally, *ism1* morphants show reduced numbers of HSPCs present in developing fish. Overall, these data indicate that *ism1* is an important gene for the formation of the embryonic zebrafish hematopoietic system.

## Materials and methods

### Zebrafish

Wildtype (AB) and transgenic zebrafish lines (*gata1a*:DsRed[57], *mpx*:EGFP[58], and *kdrl*:EGFP[59]) used in these studies were raised and maintained in accordance with California State University, Chico IACUC guidelines. All experiments were approved by the IACUC committee before being performed.

### *ism1* sequence read counts

All sequenced libraries were processed and analyzed as previously described[48].

### Generation of *ism1* mRNA

*ism1* transcript was amplified from zebrafish kidney cDNA using the following *ism1* primers: FWD 5’-ATGGTGCGTCTGGCGGCGGAG-3’ and REV 5’-TCAAAACTCCCGGGCCTCTTCA-3’. *ism1* transcript was cloned into a TOPO-TA vector (Invitrogen, Carlsbad CA) and validated by Sanger sequencing. *ism1* was than subcloned into pCS2^+^ and linearized with Not1. *ism1* mRNA was generated using a mMessage SP6 kit (Ambion, Austin, TX).

### Morpholino and *ism1* mRNA injections

*ism1* antisense morpholino (MO) was designed against the 5’ untranslated region (UTR) and start codon to prevent translation of *ism1* mRNA (Gene Tools, Philomath, OR). The MO sequence is as follows: 5’-CCAGACGCACCATCCTCTTCACC-3’. For microinjection into embryos, a mix of 8 μL of 7.6 mg/mL of *ism1* MO was mixed with 0.6 μL of phenol red for a final concentration of 7.0 ng/nL of *ism1* MO. 1 μL of this mix was loaded into a needle made with a PM102 micropipette puller (MicroData Instrument, Plainfield, NJ). Single-cell stage embryos were collected, placed onto a 1% agarose microinjection chamber plate with troughs, and injected with 1 nL (7.0 ng) of *ism1* MO with a PM 1000 Cell Microinjector (MicroData Instrument, Plainfield, NJ). For rescue injections, phenol red was reduced to 0.2 μL, and 0.4 μL of 44.7 ng/μL *ism1* mRNA was added. In this way, rescued embryos received 7.0 ng of *ism1* MO and 17.88 ng of *ism1* mRNA.

### Microscopic visualization of the hematopoietic system

To discern hematopoietic phenotypes and to quantitate cell lineages, florescent microscopy was utilized. Transgenic zebrafish were visualized under a Leica M165C (Leica, Wetzlar, Germany) fluorescent dissecting microscope at time points correlated with the emergence of specific hematopoietic cell lineages. Erythrocytes were visualized at 48 hpf with *gata1a*:DsRed transgenic animals and further examined by flow cytometry. Neutrophils and macrophages were visualized and individual cells were counted under the microscope at 48 hpf with *mpx*:GFP transgenic animals. Zebrafish and their fluorescently labeled cells were imaged using a Leica FireCam camera (Leica, Wetzlar, Germany), scored, and enumerated. For myeloid quantitation, images were labeled by a reference number and the numbers of *mpx*:GFP^+^ cells were counted in each animal by several undergraduate students to insure no bias in results.

### Flow cytometry

To enumerate the percentage of fluorescent cells in an embryo, we used transgenic zebrafish in combination with flow cytometry. 72 hpf transgenic embryos were grouped in samples of three and washed 3x with E3[60]. After the last wash, the E3 was removed, leaving 100 μL. 1 mL of 10 mM dithiothreitol (DTT) in E3 was added, and samples were incubated for 25 mins. Samples were than washed 3x with Dulbecco's phosphate-buffered saline (DPBS) containing Ca^2+^ and Mg^2+^. After the last wash, 500 μL of DPBS and 5 μL of 5 mg/mL (26U/mL) Liberase TM (Roche, Upper Bavaria, Germany) were added. Samples were incubated at 37°C on a horizontal orbital shaker at 180 rpm for 60 mins. Samples were than triturated with a P-1000 to ensure proper dissociation and transferred to a 5 mL polystyrene round bottom tube with cell strainer cap. 1 μL of SytoxRed (ThermoFisher Scientific, Waltham, MA) was added to each sample to mark dead cells. Samples were run through a BD Accuri C6 flow cytometer (BD Biosciences, San Jose, CA) and enumerated. Data were analyzed using FloJo software (FloJo LLC, Ashland, Oregon) to quantitate total percentage of positive fluorescent cells.

### Quantitation of HSPCs in developing zebrafish embryos

HSPC isolation and culture was performed as previously described[61]. Samples were given carp serum, Gcsf, and Epo to stimulate myeloid and erythroid differentiation[62, 63]. They were incubated at 32°C and 5% CO_2_ for 7–10 days and imaged with an Olympus IX53 inverted microscope (Olympus, Center Valley, PA) at 40x to enumerate colony forming units (CFUs).

### RT-PCR

mRNA was extracted from ZKS, ZEST, CHEST, and whole kidney using a Qiagen RNAeasy kit (Qiagen, Hilden, Germany). To obtain myeloid, lymphoid, and precursor cells, fluorescence-activated cell sorting (FACS) was performed on whole kidney marrow (WKM)[31]; these populations are easily separated based on their size and granularity[57]. cDNA was then generated with the iScript cDNA synthesis kit (Biorad, Hercules, CA), and PCR was performed with Jumpstart ReadyMix Taq (Sigma-Aldrich, St. Louis, MO).

### Quantitative RT-PCR (qRT-PCR)

mRNA was extracted from embryos at 48 and 72 hpf using a Qiagen RNAeasy kit (Qiagen, Hilden, Germany). cDNA was then generated with the iScript cDNA synthesis kit (Biorad, Hercules, CA), and PCR was performed with SsoFast SYBR Mastermix (Biorad, Hercules, CA). Fold expression was measured as ΔΔCT using *ef1a[28]* as a reference gene and whole kidney cDNA as a reference tissue.

### Statistics

Relative fold change was done by setting the control as the standard. For triplicates, fold change per experiments were averaged and plotted with standard deviation. To discern statistical difference, data were analyzed using an unpaired two-tailed Student’s T test. * = p<0.05, ** = p<0.0001, and N.S. = no significance.

## Results

**Fig 1:**
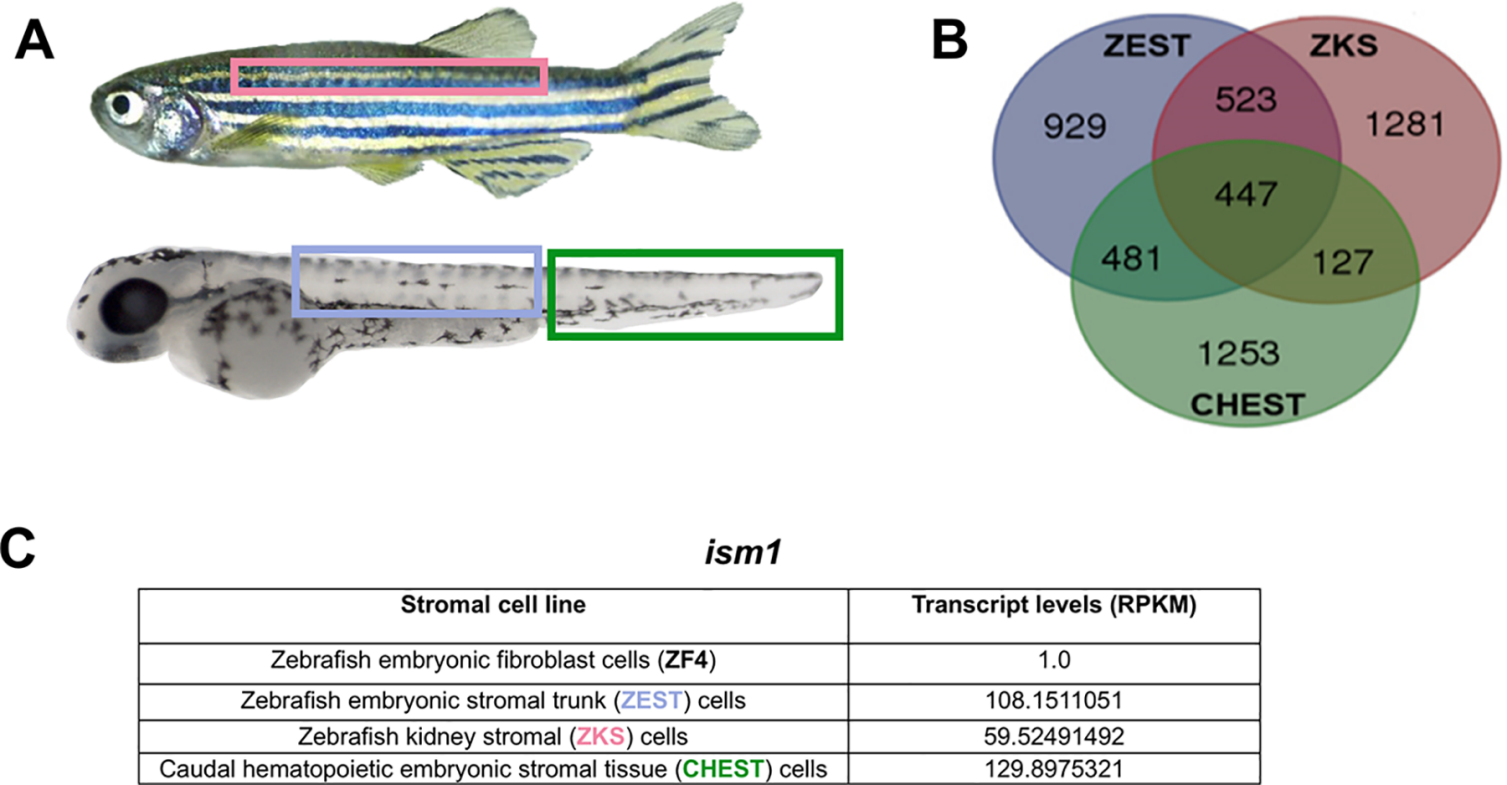
*ism1* is highly expressed in hematopoietic-supportive stromal cell lines. (**A**) Each stromal cell line is derived from sites of hematopoietic emergence, expansion, and maintenance in adult and embryonic zebrafish; adult zebrafish kidney stroma (ZKS, derived from the adult kidney; pink[50]), zebrafish embryonic stromal trunk (ZEST, derived from the embryonic trunk; blue[48]), and caudal hematopoietic embryonic stromal tissue (CHEST, derived from the CHT; green[49]). (**B**) Each stromal cell line’s transcriptome was analyzed with RNA-seq and compared to a non-hematopoietic supportive stromal cell line (ZF4); transcripts were plotted as a Venn diagram demonstrating 447 conserved transcripts. (**C**) *ism1* transcript levels for each cell line presented as reads per kilobase of transcript per million reads mapped (RPKM). See Table 1 for genes identified in this screen.

Zebrafish kidney stroma (ZKS) cells[50], zebrafish embryonic stromal trunk (ZEST) cells[48], and caudal hematopoietic embryonic stromal tissue (CHEST) cells[49] are hematopoietic-supportive stromal cell lines isolated and grown from adult and developing zebrafish. These stromal cell lines are derived from different physical locations of adult hematopoietic maintenance[64] (ZKS cells), embryonic HSC emergence[7, 8] (ZEST cells), and embryonic HSC expansion[65] (CHEST cells) at several key time points during hematopoietic development (Fig 1A) and support hematopoietic proliferation and differentiation of all blood cell types in culture[27, 48–50, 66, 67]. Therefore, we hypothesized that these cells would generate and secrete proteins required for hematopoiesis. Total RNA from each stromal cell line was isolated, sequenced, and compared to a non-supportive hematopoietic cell line (zebrafish fibroblast cells; ZF4[68]) to remove housekeeping genes from our analysis. RNA-seq of the supportive hematopoietic stromal cell lines identified 447 shared transcripts (Fig 1B; top genes involved in hematopoiesis are listed in Table 1). Transcripts were analyzed as reads per kilobase of transcript per million reads mapped (RPKM), which normalized and accounted for transcript length. From these transcripts, *ism1* was chosen for further research due to its high RPKM levels across all three cell lines (Fig 1C), the fact that it is a secreted protein, and because it has a conserved ortholog in humans. To confirm that *ism1* was expressed in ZKS, ZEST, and CHEST stromal cells, we performed RT-PCR for *ism1*, validating its expression in these tissues (S1 Fig). We also observed *ism1* expression in whole kidney, the main site of hematopoiesis in the adult zebrafish[64]. To confirm that *ism*1 was only expressed in the stromal cells of the kidney, we fractionated cells from WKM using FACS; myeloid, lymphoid, and precursor cell populations can be easily separated based on their size and granularity[57]. RT-PCR analysis of these cells confirmed that *ism1* is expressed in the supportive hematopoietic tissues, but not in hematopoietic cells themselves (S1 Fig).

**Table 1:** Expression levels of highly expressed transcripts in hematopoietic supportive cell lines.

Gene ID (Zv9)	Gene name	Gene description	RPKM (ZKS cells)	RPKM (ZEST cells)	RPKM (CHEST cells)
ENSDARG00000056680	*stc2a*	Stanniocalcin 2a	1171.390702	1231.42446	706.1714178
ENSDARG00000015050	*calm3a*	Calmodulin 3a (phosphorylase kinase, delta)	398.2576181	445.6855878	969.0665779
ENSDARG00000026726	*anxa1a*	Annexin A1a	386.1286169	280.2930981	417.0527221
ENSDARG00000071498	*fhl1a*	Four and a half LIM domains 1a	392.740221	209.8129055	350.3135956
ENSDARG00000055439	*adamtsl7*	ADAMTS-like 7	377.7311012	296.1991765	268.6541787
ENSDARG00000069752	*ckba*	Creatine kinase, brain a	410.942751	156.7429014	276.7297589
ENSDARG00000016691	*cd9b*	CD9 antigen, b	506.2882968	266.2312285	66.00918816
ENSDARG00000093440	*tnfaip6*	Tumor necrosis factor, alpha-induced protein 6	197.4394341	109.3081398	491.6323329
ENSDARG00000038123	*myl9a*	Myosin, light chain 9a, regulatory	179.6584981	96.56364658	511.0645904
ENSDARG00000014626	*dlx3b*	Distal-less homeobox gene 3b	285.6044597	300.5094173	166.0309536
ENSDARG00000087402	*tpm1*	Tropomyosin 1 (alpha)	147.8702844	67.17783708	530.8673917
ENSDARG00000029353	*serpine2*	Serine (or cysteine) proteinase inhibitor, clade E (nexin, plasminogen activator inhibitor type 1), member 2	155.1438879	185.8865435	362.6601572
ENSDARG00000008359	*cnn3a*	Calponin 3, acidic a	200.6466151	175.5868376	322.1817671
ENSDARG00000013804	*capns1b*	Calpain, small subunit 1 b	295.8800252	192.2972637	137.1191417
ENSDARG00000062363	*phex*	Phosphate regulating gene with homologues to endopeptidases on the X chromosome	374.7902084	246.0473474	2.923096804
ENSDARG00000007697	*fabp7a*	Fatty acid binding protein 7, brain, a	195.9918097	237.0929702	156.4171196
ENSDARG00000054749	*lmo4b*	LIM domain only 4b	171.4754089	158.0390988	259.5479538
ENSDARG00000040362	*ehd2*	EH-domain containing 2	161.7766861	245.0843826	179.9114927
ENSDARG00000012066	*dcn*	Decorin	138.738941	110.0299673	294.5159175
ENSDARG00000007396	*rnd3b*	Rho family GTPase 3b	230.919067	106.1477521	180.3513591
ENSDARG00000075045	*cxcl-c1c*	Chemokine (C-X-C motif) ligand C1c	205.5342189	222.3706168	85.84923297
ENSDARG00000030449	*crabp2b*	Cellular retinoic acid binding protein 2, b	105.0984741	221.8625863	181.7852462
ENSDARG00000089187	*wfdc2*	WAP four-disulfide core domain 2	215.3192844	100.2819887	173.5050759
ENSDARG00000061226	*timp2a*	Tissue inhibitor of metalloproteinase 2a	110.5132145	134.7435353	217.234448
ENSDARG00000030694	*atp6v1e1b*	ATPase, H+ transporting, lysosomal, V1 subunit E isoform 1b	211.5850639	104.0432062	140.8175355
ENSDARG00000016238	*arl6ip5b*	ADP-ribosylation factor-like 6 interacting protein 5b	180.1080903	105.0459892	159.1043514
ENSDARG00000019307	*dusp5*	Dual specificity phosphatase 5	126.478358	236.8883821	68.27162288
ENSDARG00000054823	*id3*	Inhibitor of DNA binding 3	123.0298414	119.957024	185.8068645
ENSDARG00000096118	*ost4*	Oligosaccharyltransferase 4 homolog	293.697141	56.14183101	47.90212635
ENSDARG00000070391	*tspan4b*	Tetraspanin 4b	124.4627775	134.439824	107.2748044
ENSDARG00000002507	*itga10*	Integrin, alpha 10	89.61611629	201.1502394	71.71587866
ENSDARG00000008363	*mcl1b*	Myeloid cell leukemia sequence 1b	138.0029954	100.3628761	121.8545135
ENSDARG00000078674	*hspb9*	Heat shock protein, alpha-crystallin-related, 9	208.1065389	130.9142075	9.6011956
ENSDARG00000056627	*cxcl14*	Chemokine (C-X-C motif) ligand 14	30.54406211	119.5273691	197.7629774
ENSDARG00000026070	*cd82b*	CD82 antigen, b	117.1867309	119.7501676	99.79403009
ENSDARG00000032831	*htra1a*	HtrA serine peptidase 1a	62.04851894	79.54294838	191.2823828
ENSDARG00000027088	*ptgdsb*	Prostaglandin D2 synthase b	295.7620764	16.83617366	7.299828587
ENSDARG00000069983	*scinla*	Scinderin like a	229.2763242	77.71766924	12.73139596
ENSDARG00000014103	*dkk1a*	Dickkopf 1a	45.36030464	95.82598311	174.3537947
ENSDARG00000044001	*lgals3l*	Lectin, galactoside-binding, soluble, 3 (galectin 3)-like	151.485882	94.79980323	68.69664737
ENSDARG00000094752	*rpe65b*	Retinal pigment epithelium-specific protein 65b	51.81279893	157.9199935	85.8643745
ENSDARG00000006275	*irf2bp2b*	Interferon regulatory factor 2 binding protein 2b	83.23427126	90.32706729	118.4504944
ENSDARG00000096594	*lratb*	Lecithin retinol acyltransferase b (phosphatidylcholine—retinol O-acyltransferase b)	54.62075299	126.7544822	101.4833434
ENSDARG00000045219	*dkk1b*	Dickkopf 1b	115.2674028	123.3601222	41.36781122
ENSDARG00000073891	*gdf10b*	Growth differentiation factor 10b	33.67260166	219.1972506	6.710000293
ENSDARG00000063704	*gpr1*	G protein-coupled receptor 1	11.47967914	67.08867022	174.2527408
ENSDARG00000042690	*s1pr1*	Sphingosine-1-phosphate receptor 1	51.05707689	120.3548169	69.24951973
ENSDARG00000070420	*cyp24a1*	Cytochrome P450, family 24, subfamily A, polypeptide 1	18.72705544	177.0835255	44.13670934
ENSDARG00000086842	*dap1b*	Death associated protein 1b	108.3112632	77.42847935	53.19516114
ENSDARG00000068710	*nid1a*	Nidogen 1a	62.19315736	114.8440231	50.83336553
ENSDARG00000055381	*bambia*	BMP and activin membrane-bound inhibitor homolog a	88.97243166	57.43071367	78.67391423
ENSDARG00000067742	*eva1bb*	Eva-1 homolog Bb	63.44368564	84.2765728	74.97828951
ENSDARG00000005789	*enpp1*	Ectonucleotide pyrophosphatase/ phosphodiesterase 1	64.34284892	137.6726653	19.82855613
ENSDARG00000056023	*hoxb9a*	Homeo box B9a	69.19604476	83.45231228	68.31730246
ENSDARG00000094857	*dio2*	Deiodinase, iodothyronine, type II	79.95831483	112.2283984	26.87641183
ENSDARG00000089078	*col23A1*	Collagen, type XXIII, alpha 1	63.62339938	147.7007832	2.289688031
ENSDARG00000030177	*uchl3*	Ubiquitin carboxyl-terminal esterase L3 (ubiquitin thiolesterase)	105.7558676	48.45357406	49.98198164
ENSDARG00000024877	*ptgr1*	Prostaglandin reductase 1	103.6331577	54.73419065	37.55761808
ENSDARG00000094854	*ms4a17a*.*8*	Membrane-spanning 4-domains, subfamily A, member 17A.8	116.1375676	55.21694763	23.36757024
ENSDARG00000019128	*tpm4b*	Tropomyosin 4b	54.49279439	41.58138656	98.05974096
ENSDARG00000058348	*scinlb*	Scinderin like b	43.90721933	56.88955333	90.22067671
ENSDARG00000005541	*wif1*	Wnt inhibitory factor 1	93.47095208	67.6434638	28.05685047
ENSDARG00000010462	*sp9*	Sp9 transcription factor	57.92009712	49.7884664	76.65945541
ENSDARG00000044803	*dhrs3b*	Dehydrogenase/reductase (SDR family) member 3b	130.0154057	51.68314191	1.624036879
ENSDARG00000087263	*ssr3*	Signal sequence receptor, gamma	91.92315352	47.64499378	39.03838439
ENSDARG00000058733	*ihha*	Indian hedgehog homolog a	7.794542694	88.86938529	78.16679263
ENSDARG00000015966	*yaf2*	YY1 associated factor 2	67.3993785	69.0931997	38.15094538
ENSDARG00000090945	*clec4f*	C-type lectin domain family 4, member F	120.1210729	48.41536445	3.207217092
ENSDARG00000042296	*dlx5a*	Distal-less homeobox gene 5a	126.8385026	31.88420836	12.30766798
ENSDARG00000053381	*ppap2a*	Phosphatidic acid phosphatase type 2A	36.20007779	67.05858162	65.57173276
ENSDARG00000069376	*tnfsf12*	Tumor necrosis factor (ligand) superfamily, member 12	38.06281765	121.2490869	7.954191675

All transcripts are presented as reads per kilobase of transcript per million reads mapped (RPKM). Each stromal cell line’s transcriptome was analyzed and compared to a non-hematopoietic supportive stromal cell line (ZF4). The transcripts below have been described previously as playing a role in hematopoiesis.

### *ism1* morphants exhibit reduced erythrocyte production

**Fig 2:**
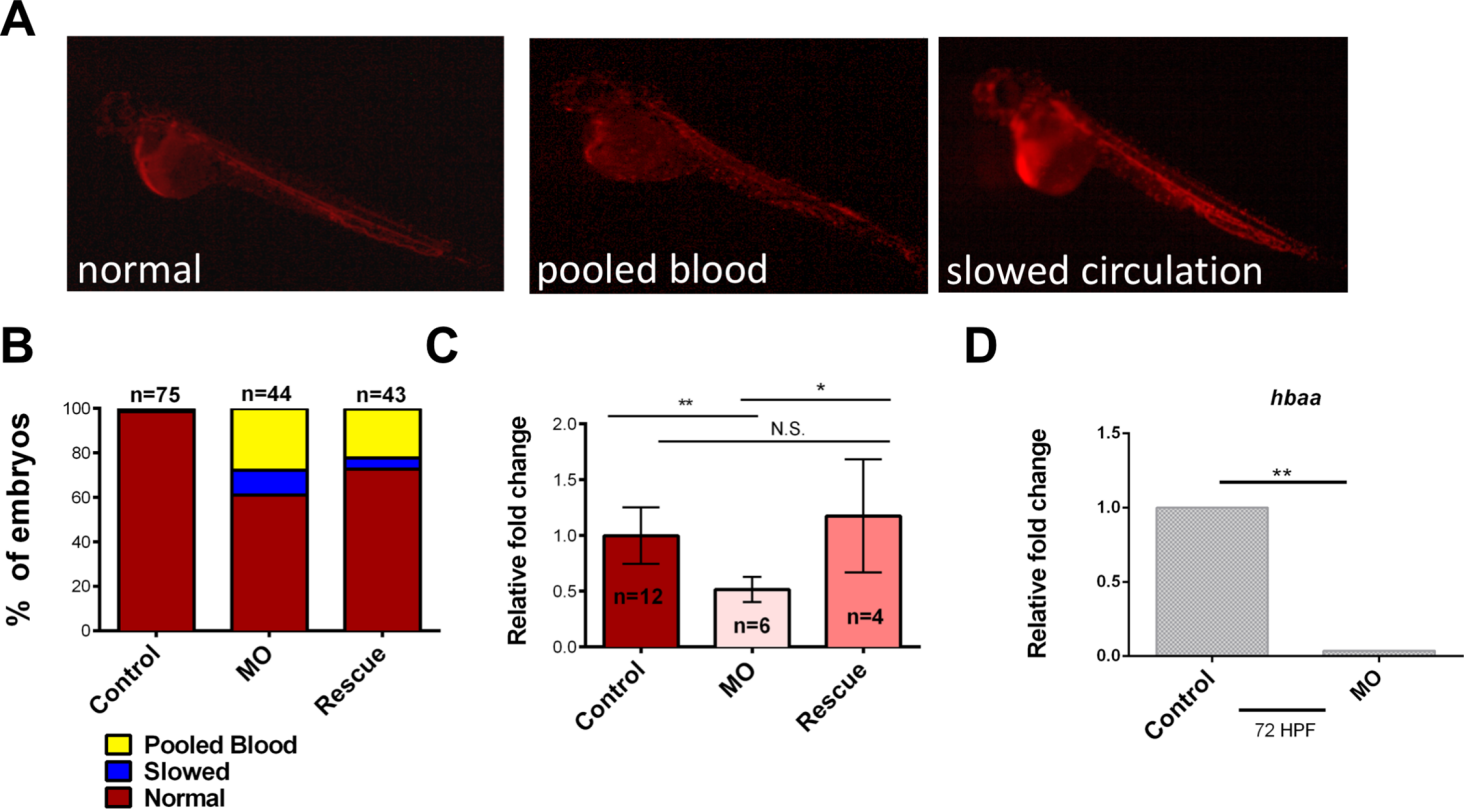
*ism1* knockdown decreases erythrocytes. *gata1*:DsRed zebrafish embryos were injected at the single-cell-stage with 7 ng *ism1* MO (MO), or 7 ng *ism1* MO and 17.88 ng of *ism1* mRNA (Rescue); uninjected embryos served as controls. (**A**) 24 hpf zebrafish visualized at 5x that have normal (left), pooled blood (middle), and slowed circulation (right) phenotypes. (**B**) Quantitation of zebrafish shown in (**A**); pooled blood (yellow), slowed circulation (blue), and normal (red) phenotypes. (**C**) Flow cytometry results quantitating *gata1*:DsRed^+^ erythrocytes in uninjected (control; red), *ism1* MO injected (MO; peach), and *ism1 MO* with *ism1* mRNA (Rescue; pink) 72 hpf zebrafish. (**D**) qRT-PCR was performed comparing transcript levels of *hbaa* in *ism1* MO injected (MO) and uninjected controls (control). Bars represent the mean, and error bars represent standard deviation. Data was normalized as fold change over control and analyzed using a two-tailed unpaired Student’s T test. * denotes p<0.05, ** denotes p<0.0001, N.S indicates no significance.

To elucidate *ism1’s* role in erythroid cell production, *gata1a*:DsRed transgenic zebrafish were used; Gata1 is a transcription factor expressed in erythrocytes, allowing for real time visualization of red blood cells in developing embryos. *ism1* knockdown with a specific MO caused slowed circulation and caused blood to pool in *gata1a*:DsRed^+^ zebrafish (Fig 2A) when examined at 48 hpf. The control group expressed no abnormalities with respect to blood circulation, compared to 38% of the *ism1* morphants. The hearts of embryos injected with *ism*1 MO were pumping and appeared morphologically normal. Additionally, no blood was observed leaking from blood vessels. Injection of *ism1* mRNA reduced the amount of pooled blood seen in *ism1* morphants and increased the speed of erythrocyte circulation (Fig 2B).

Regardless of their circulation phenotypes, we speculated that the morphant embryos also had decreased numbers of erythrocytes. To quantitate total red blood cell numbers, flow cytometry was utilized. All *ism1* MO injected embryos were analyzed without grouping them based on their circulation phenotype. Three individual 72 hpf *gata1a*:DsRed^+^ *ism1* MO injected zebrafish were randomly grouped together, enzymatically dissociated in a sample tube, and analyzed on a flow cytometer to quantitate *gata1a*:DsRed^+^ cells. Percentages of *gata1a*:DsRed^+^ cells were normalized to uninjected control embryos and expressed as fold change over control. *ism1* knockdown caused an approximate 2-fold decrease in *gata1*:DsRed^+^ cells compared to control (Fig 2C) and the rescued group displayed erythrocyte levels similar to control groups (Fig 2C), indicating that *ism1* depletion was the cause of the erythrocyte defect. Additionally, *ism1* MO injected whole embryos were pooled into groups of 10, digested, and qRT-PCR was used to analyze alpha globin *(hbaa)* mRNA levels, a specific marker of differentiated erythrocytes. qRT-PCR demonstrated a 29-fold reduction of *hbaa* mRNA levels in *ism1* MO injected animals (Fig 2D). Taken together, these data indicate that *ism1* reduction causes a decrease in embryonic erythrocytes and a decrease in *hbaa* mRNA levels in the whole organism.

While intersegmental vessels (ISVs) are not involved in blood development, previous studies indicated that *ism1* was necessary for proper development of ISVs in zebrafish[55]. To confirm that *ism1* was not necessary for the formation of the dorsal aorta, the source of HSC generation during development, we injected *ism1* MO into the vasculature-specific *kdrl*:EGFP zebrafish transgenic line. 24 hpf zebrafish were visualized and exhibited shortened ISVs, as reported previously (S2 Fig)[55]. However, the dorsal aorta was not negatively affected in morphants (S2 Fig), also in agreement with previous studies[55]. Although not previously described, the shortened ISVs recovered by 48 hpf without addition of exogenous *ism1*. In conclusion, *ism1* morphants experience a reduction in ISVs that recovers by 48 hpf but have a normal dorsal aorta.

### *ism1* morphants exhibit reduced myeloid cells

**Fig 3:**
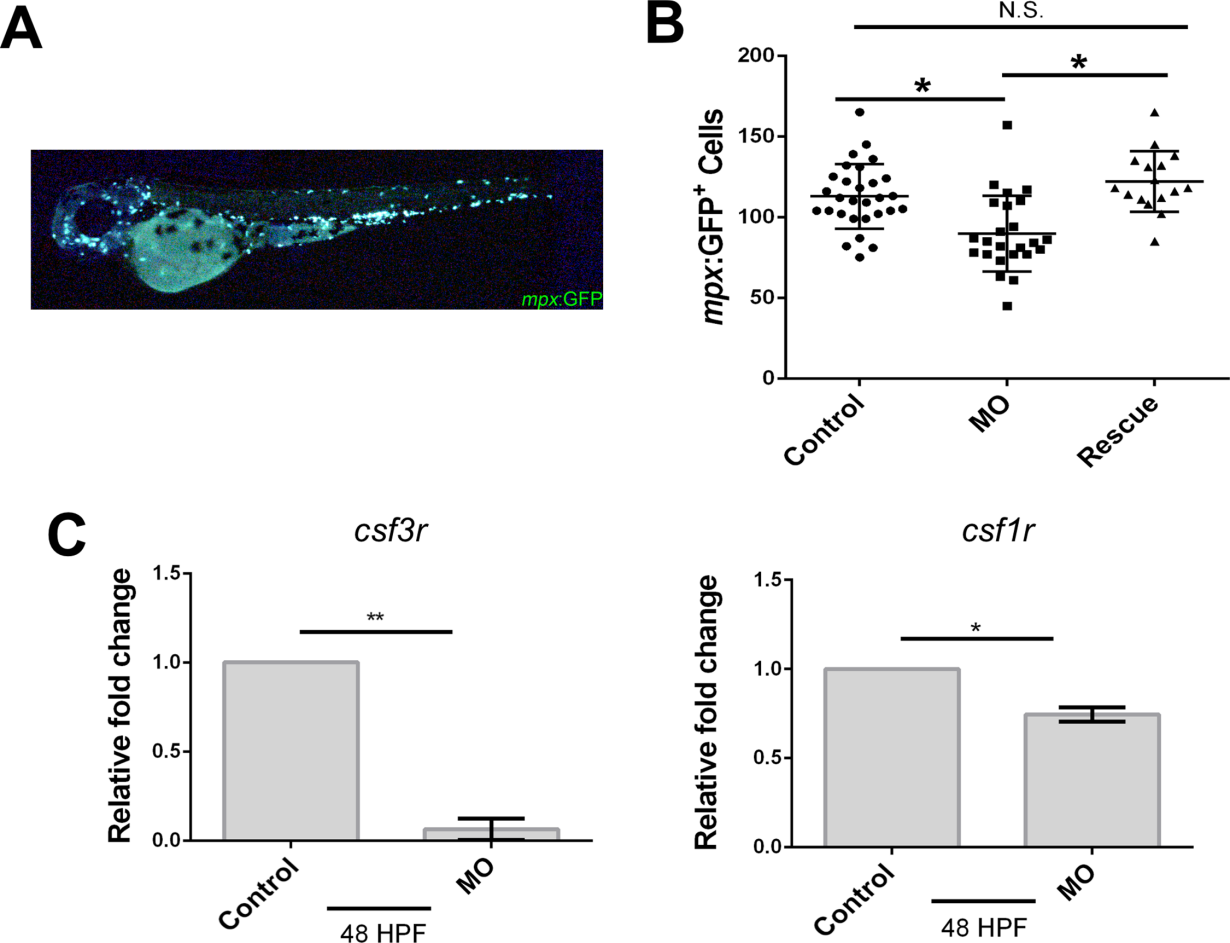
*ism1* knockdown decreases neutrophils and macrophages in 48 hpf zebrafish. *mpx*:GFP single-cell-stage embryos were injected with 7 ng *ism1* MO (MO; squares), or 7 ng *ism1* MO and 17.88 ng of *ism1* mRNA (Rescue; triangles); uninjected embryos served as controls (circles). (**A**) Developing 48 hpf zebrafish were visualized, and individual *mpx*:GFP^+^ cells were enumerated; representative fish shown for reference. (**B**) Each data point represents total amount of *mpx*:GFP^+^ cells present in one zebrafish. (**C**) qRT-PCR was performed comparing transcript levels of *csf3r* (left) and *csf1r* (right) in *ism1* MO injected (MO) and uninjected controls (control) at 48 hpf. Bars represent the mean, and error bars represent standard deviation. A two-tailed unpaired Student’s T test was performed to determine statistical significance. * denotes p<0.05, ** denotes p<0.0001, N.S indicates no significance.

Myeloid cells such as neutrophils and macrophages are cellular components critical for innate immune responses. To investigate *ism1*’s role in myeloid cell biology, *mpx*:EGFP transgenic zebrafish were utilized. *mpx*:EGFP zebrafish embryos were injected with *ism1* MO; all injected embryos were visualized and total *mpx*:EGFP^+^ cells were individually enumerated at 48 hpf (Fig 3A). *ism1* MO injection resulted in a 20% reduction of *mpx*:EGFP^+^ cells compared to control groups (Fig 3B) and rescue with *ism1* mRNA showed a recovery of *mpx*:EGFP^+^ cells compared to *ism1* MO injected embryos. qRT-PCR was performed on pools of 10 injected embryos for colony stimulating factor 3 receptor (*csf3r;* also called granulocyte colony stimulating factor receptor), and colony stimulating factor 1 receptor (*csf1r;* also called macrophage colony stimulating factor receptor), two markers for neutrophils and macrophages, respectively. qRT-PCR indicated a 11-fold reduction in *csf3r* mRNA levels and a 30% reduction of *csf1r* mRNA levels in *ism1* MO injected embryos (Fig 3C). Overall, these data indicate that *ism1* knockdown causes a reduction in *mpx*:EGFP^+^ myeloid cells.

### HSPCs are reduced in *ism1* morphants

**Fig 4:**
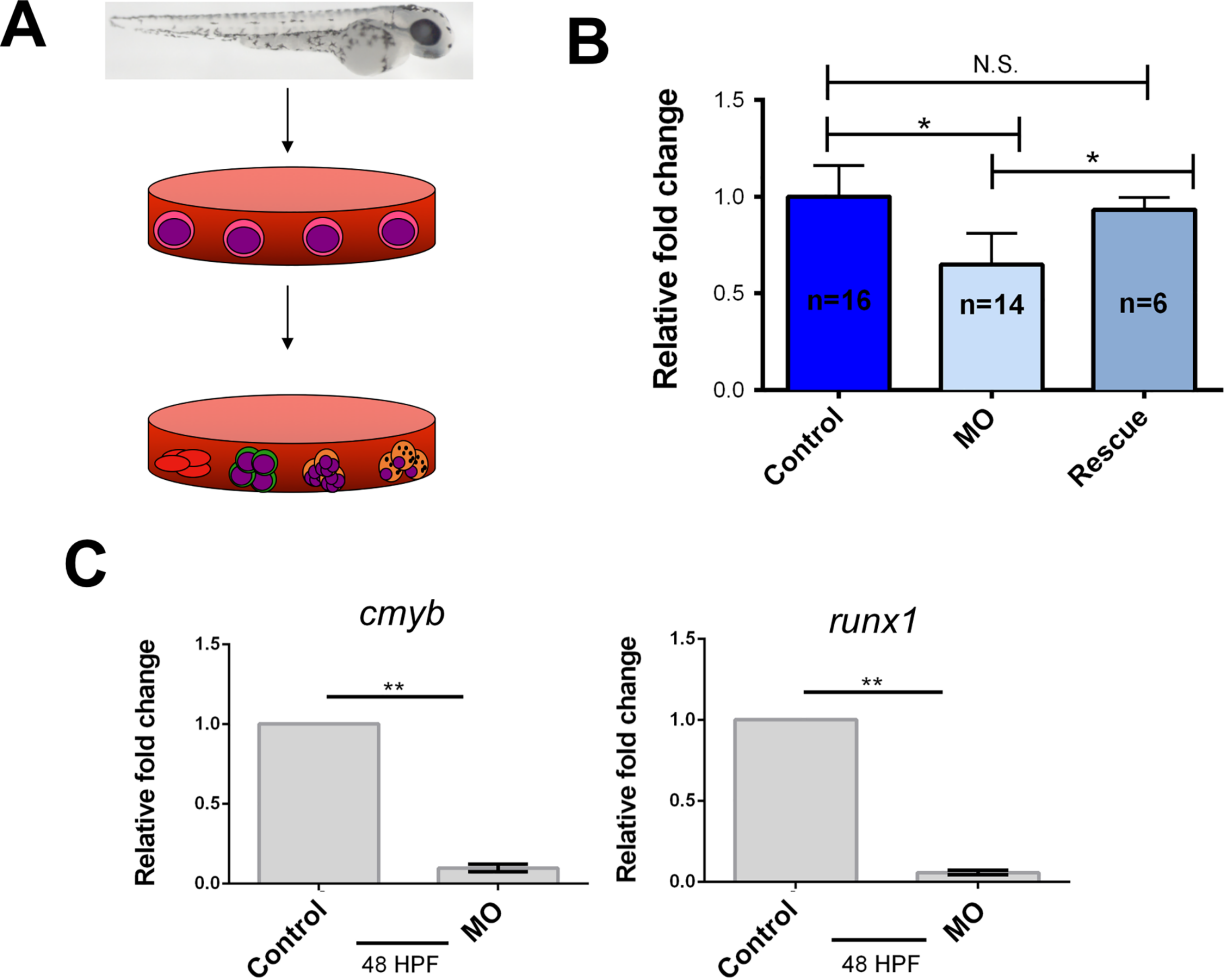
Hematopoietic stem and progenitor cells (HSPCs) are reduced in *ism1* knockdown embryos. Single-cell-stage embryos were injected with 7 ng of *ism1* MO (MO), or 7 ng of *ism1* MO and 17.88 ng of *ism1* mRNA (Rescue); uninjected embryos served as a control. (**A**) Ten 48 hpf embryos from each treatment were digested, plated in methylcellulose with exogenous hematopoietic-supportive growth factors, and incubated for 7 days. (**B**) Colony forming units (CFUs) generated by *ism1* MO (light blue) and *ism1* MO with *ism1* mRNA (Rescue; darker blue) injected embryos presented as fold change over control embryos (dark blue). (**C**) qRT-PCR was performed comparing transcript levels of *cmyb* and *runx1* in *ism1* MO injected (MO) and uninjected controls (control) at 48 hpf. Bars represent the mean, and error bars represent standard deviation. A two-tailed unpaired Student’s T test was performed to determine statistical significance. * denotes p<0.05, ** denotes p<0.0001, N.S indicates no significance.

To confirm that *ism1* loss was causing a reduction in HSPCs, groups of 10 MO-injected embryos at 48 hpf were placed in a tube, enzymatically digested, and plated in methylcellulose with exogenous hematopoietic-supportive growth factors[61]. After plating, the embryos were incubated for 7 days, allowing HSPCs to proliferate and differentiate (Fig 4A). Fish were not phenotypically selected before digestion; all injected fish were randomly pooled into groups of 10 and plated in methylcellulose. *ism1* MO injected and rescued embryos generated hematopoietic colonies, which were enumerated. Colony forming units (CFUs) for *ism1* MO injected zebrafish were reduced by 35% compared to control and rescue groups (Fig 4B). There was no statistical difference between control and rescue groups, indicating that *ism1* is necessary for the generation of the proper numbers of HSPCs in developing zebrafish embryos. qRT-PCR on 48 hpf embryos was also performed to quantitate HSPC-related transcription factors. Data showed an overall 20-fold reduction in *runx1*, and a 10-fold reduction in *cmyb* mRNA levels between controls and *ism1* MO injected embryos (Fig 4C). Overall, these data indicate that HSPC formation is reduced in *ism1* morphants.

## Discussion

In this study, we sought to elucidate molecular requirements for HSPC generation, support, and expansion. Utilizing previously described zebrafish hematopoietic-supportive stromal cell lines[48–50] we identified highly expressed transcripts determined by RNA-seq. *ism1* was chosen from this list due to its involvement in angiogenesis[55, 69], its presence in embryonic[52, 54, 55] and adult[53, 54] hematopoietic tissue, the fact that it is a secreted protein[52, 53, 55], and because it shares synteny with human *ISM1*. Our study findings indicate that *ism1* is required for proper embryonic hematopoiesis.

To elucidate *ism1*’s role in hematopoiesis we performed loss-of-function experiments using MOs to generate *ism1* morphants. Other MOs have been utilized to study *ism1*[55], and our MO was directed towards a similar location in the genome 8bp 3’ of Xiang *et*. *al’s* translation-blocking MO; the location we chose was suggested by modeling software to be a more effective site for blocking *ism1* translation. The MO we designed binds 10bp 5’ to *ism1*’s start site; as two splice variants are predicted for *ism1*, we wanted to ensure that the MO blocked all versions of Ism1 so we would not have multiple splice isoforms potentially confounding our results. Importantly, our MO and Xiang *et*. *al*’s have the same phenotypic effect on ISV formation early in development. The dorsal aorta, the site of HSPC generation between 36–52 hpf[7, 8], was unaffected (our data and [55]). We also observed that ISVs were only affected between 24–48 hpf, after which ISV branching was normal. To observe hematopoietic defects, we injected *ism1* MOs into previously characterized transgenic fish lines that have fluorescent proteins expressed in specific hematopoietic cell lineages. *ism1* morphants did not express any morphological defects such as bent tails, spinal deformation, or stunted growth (data not shown). However, they did have phenotypes in their hematopoietic tissues. Coupled with the fact that these phenotypes were rescued by the addition of exogenous *ism1* mRNA indicates that Ism1 is a key protein involved in normal zebrafish hematopoiesis.

It is important to note that we are utilizing zebrafish as a model system to investigate the evolution of vertebrate hematopoiesis. While mammals are derived from lobe-finned fish and zebrafish are ray-finned fish (reviewed in [70]), zebrafish have proven to be an excellent model of hematopoiesis; their molecular control of blood formation and differentiation is well conserved with mammals[24–26, 71]. Further evidence of this is that drugs identified in zebrafish are now being utilized in the clinic to treat human blood diseases[72]. One concern with using zebrafish as a model is that the MO may not be specific for only one copy of *ism1*. While the genome was duplicated early in teleost evolution[73, 74], there is only one described copy of *ism1* in the zebrafish genome; we found no additional copies in our scanning of genomic data and RNA-seq results, in agreement with others[55]. This one copy of zebrafish *ism1* shares synteny with human *ISM1*; *JAG1* (*jag1b*), *BTBD3* (*btbd3b*), and *SPTLC3* (*sptlc3*) are 5’ to *ISM1* (*ism1*), while *TASP1* (*tasp1*), *KIF16B* (*kif16bb*), and *FLRT3* (*flrt3*) are 3’. The only other genes in the zebrafish *ism1* family are *ism2a* and *ism2b*. These two *isthmin* paralogs are located on different chromosomes (*ism1* is on chromosome 13, *ism2a* is on 17, and *ism2b* is on 20) and share synteny with only the genes on the 5’ side of human *ISM2*. Little is known about *ism2a* or *ism2b*’s roles/functions in zebrafish. These genes share no synteny with *ISM1* or *ism1*, indicating that *ism2a* and *ism2*b are likely different genes as opposed to duplicates of *ism1*. There are 9 *ism1* orthologs in other fish species like cod, amazon molly, fugu, medaka, platyfish, spotted gar, stickleback, tetraodon, and tilapia, all of which only have one identified copy of *ism1* in their genome. Overall, it appears that *ism1* only has one identifiable copy in the zebrafish genome, is not mislabeled, and is similar to human *ISM1*.

To examine the role of *ism1* in hematopoiesis, we first examined erythrocytes due to their abundance and importance during development; they are also the first hematopoietic cells to arise. *gata1a*:DsRed^+^ *ism1* morphants exhibited pooled blood and we observed what appeared to be slowed circulation. While some “pooled blood” phenotypes have been attributed to functional cardiac defects[75], no such phenotype was visualized in our experiments. This suggested that the morphant’s vasculature might be disrupted, but our data, in agreement with prior studies[55], did not see a disruption of the dorsal aorta after *ism1* knockdown. To further confirm that erythrocytes were specifically reduced, we enumerated them with flow cytometry and found a significant decrease in erythrocytes in *ism1* morphants. Reduction of erythrocytes was further validated by qRT-PCR data showing reduction of the *hbaa* transcript, a hemoglobin gene. In conclusion, *ism1* knockdown caused an overall reduction of erythrocytes and the phenotype was rescued by the addition of exogenous *ism1* mRNA, indicating that *ism1* is required for normal erythropoiesis.

*mpx*:GFP zebrafish express GFP in both neutrophils and macrophages[58, 63]; both are differentiated myeloid cell lineages. *ism1* morphants had a 20% reduction in these myeloid cells, suggesting that myeloid cell lineages were not properly forming. We also saw a reduction in *csf3r* transcript, the signaling receptor necessary for granulocyte formation and differentiation, measured by qRT-PCR. While *ism1* morphants exhibited an overall decrease in myeloid cells, these cell lineages were still generated, suggesting that *ism1* isn’t required for myeloid differentiation but is necessary for the generation or proliferation of myeloid HSPCs. Together, the reduction in both myeloid and erythroid cell lineages suggested a reduction of a common upstream progenitor cell. Therefore, *ism1* is likely involved in the proliferation or differentiation of a multipotent progenitor such as the CMP or HSC.

Together, decreased production of differentiated erythroid and myeloid cell lineages indicated that *ism1* knockdown somehow decreased the generation or proliferation of HSPCs during development. To investigate this possibility, we developed a surrogate assay to assess HSPC numbers in morphant zebrafish embryos[61]. This assay involved plating dissociated whole zebrafish embryos and adding exogenous factors that promote only HSPC differentiation and proliferation *in vitro*[62, 63, 76]. Plating HSPCs generates CFUs that arise from a single progenitor cell. Therefore, the number of CFUs counted after 7 days in culture corresponds to the number of total progenitor cells present in the organism at the time they were assayed. These results suggest that *ism1* is transcribed, translated, and secreted by stromal cells during a window of HSPC formation in the embryo; reducing *ism1* expression during this time reduced the number of HSPCs in the developing zebrafish. This non-cell-autonomous effect of *ism1* is seen with the clonal methylcellulose assay- because less Ism1 is secreted by the hematopoietic stroma during development, less HSPCs are generated in the embryo. 48 hpf was chosen for these assays due to the fact that functional HSPCs are generated by this time in development[7, 8, 27–30]. Our data, in agreement with previous studies[54], indicates that *ism1* is expressed in the hematopoietic stroma, but not in hematopoietic cells themselves. These results reinforce that Ism1 is a secreted protein made in the stromal niche, and not produced by HSPCs themselves; HSPCs are present in the “precursor”[62, 63, 76, 77] and “lymphoid”[29, 57, 62, 63, 76, 77] fractions of blood cells analyzed in this study. Importantly, this was not a developmental delay; plating dissociated *ism1* morphant embryos at later time points did not rescue the number of HSPC-derived colonies present (data not shown). Overall, the fact that HSPCs are reduced in *ism1* morphants likely accounts for the decreased counts of differentiated erythroid and myeloid lineages, suggesting that *ism1* is needed for normal levels of HSPC formation and differentiation during embryonic development.

*ism1* is extremely enigmatic, and many seemingly conflicting roles for this gene exist in the literature. *ism1* was originally identified as a secreted protein expressed along with fibroblast growth factor (Fgf) 8 in the *Xenopus* midbrain-hindbrain organizer[52], and it has recently been described in craniofacial patterning in humans[78]. Importantly, it has been identified in multiple embryonic tissues, many of which are involved in various regions of embryonic development. Of interest for blood formation, *ism1* was identified in *Xenopus* blood islands[52] and the lateral plate mesoderm of chicks and mice[54], which is tissue that gives rise to blood. It is also found in bone marrow[54], the site of adult hematopoiesis in mammals. While *ism1* was identified in the supportive hematopoietic stroma and not actual blood cells (our data and [54]), other studies indicate Ism1 is expressed in specific subsets of mouse lymphoid cells, including T and natural killer (NK) cells[53]. Our data does not correlate with this; zebrafish T and NK cells are present in the “lymphoid” population of WKM[57], and we detected no *ism1* in this fraction, indicating that this may be a difference between fish and mammals. Another difference across species is the description of *ism1* as an angiogenesis inhibitor; in mice exogenous ISM1 suppressed tumor angiogenesis[55, 69], but in zebrafish the knockdown of *ism1* reduced the growth of intersegmental vessels[55]. While *Ism1* plays a role in apoptosis, its role in this process is also complicated. It specifically reduces endothelial cell (EC) growth by inducing apoptosis[55, 69], while it has little to no effect on the proliferation or survival of glioma cells[69], NIH3T3 fibroblasts[55], Swiss 3T3 fibroblasts[55], B16 melanoma cells[55], and hepatocellular carcinoma cells[55]. This differential apoptotic capacity is not just different across cell types; it is linked to the solubility of ISM1. Soluble ISM1 causes apoptosis, while extracellular matrix (ECM)-immobilized ISM1 promotes EC adhesion, migration, and survival[79]. Unfortunately, no zebrafish-specific Ism1 antibody exists, preventing analysis of this in our experiments. It is important to note that the role of Ism1-induced apoptosis of hematopoietic cells is a possibility that could also explain a reduction in blood cells; further investigation may shed light on this subject.

Overall, these findings demonstrate a new role for *ism1;* its involvement in *de novo* hematopoietic cell formation. *ism1* reduction negatively impacted the number of HSPCs formed and their ability to properly generate mature erythrocytes, neutrophils, and macrophages. This could be due to *ism1* affecting a variety of signaling pathways; we speculate that *ism1* is involved in molecular pathways that instruct HSPC formation and differentiation, as its knockdown decreases various mature blood cell lineages. This is of keen interest, because *ism1* is secreted by our hematopoietic-supportive cell lines and affects various cell lineages, yet it has only been identified in a handful of tissues[52–55, 78] and lymphoid cells[53]. *ism1* expression is present in *Xenopus* blood islands suggesting its role in generating primitive blood[52]. This expression also implicates its importance in definitive hematopoiesis, as primitive erythropoiesis expresses many of the same molecular factors as its definitive counterpart[80]. *ism1* is also expressed at the same time and in the same location as *Xenopus* Fgf8 in the notochord[52]; it is also present in the zebrafish notochord[55]. FGF signaling is involved in hematopoiesis[81–87] as it is a mediator of Notch and Wnt signaling[83]. It is also modulated by BMP signaling[82]. All of these pathways are required for HSC production. Interestingly, *ism1* is a Wnt target[56] that is expressed (and likely secreted from) the notochord[52, 55]; this is the first time a secreted signal from the notochord has been implicated in hematopoietic development. Overall, these previous studies and our results indicate *ism1* is signaling to induce formation and functionality of hematopoietic tissues in the developing zebrafish. Further investigation of *ism1*’s role in this process may shed light on an important signaling pathway for blood formation and regulation.

## Supporting information

S1 Fig *ism1* is expressed in hematopoietic-supportive stromal cells but not in hematopoietic cells. RT-PCR was performed for *ism1* from ZKS, ZEST, CHEST, and kidney mRNA. Myeloid, lymphoid, and precursor cell mRNA isolated from zebrafish kidney was also interrogated for *ism1* transcripts.
(TIF)

S2 Fig *ism1* knockdown leads to temporally shortened intersegmental vessels. *flk1*:GFP single-cell-stage embryos were injected with 7 ng of *ism1* MO (bottom); uninjected embryos served as controls (top). 24 hpf (left column) and 48 hpf (right column) zebrafish were visualized at 40x for *flk1*:GFP fluorescence within the trunk area denoted by black box in brightfield image at top center. Arrows indicate shortened intersegmental vessels in *ism1* morphants. Numbers in corner of images denote the number of embryos displaying the imaged phenotype.
(TIF)
