# Supplementary figures and images for: Isthmin 1 (ism1) is required for normal hematopoiesis in developing zebrafish
Source: PLoS One. 2018 May 14;13(5):e0196872. doi: 10.1371/journal.pone.0196872 (PMC5951578; doi:10.1371/journal.pone.0196872)

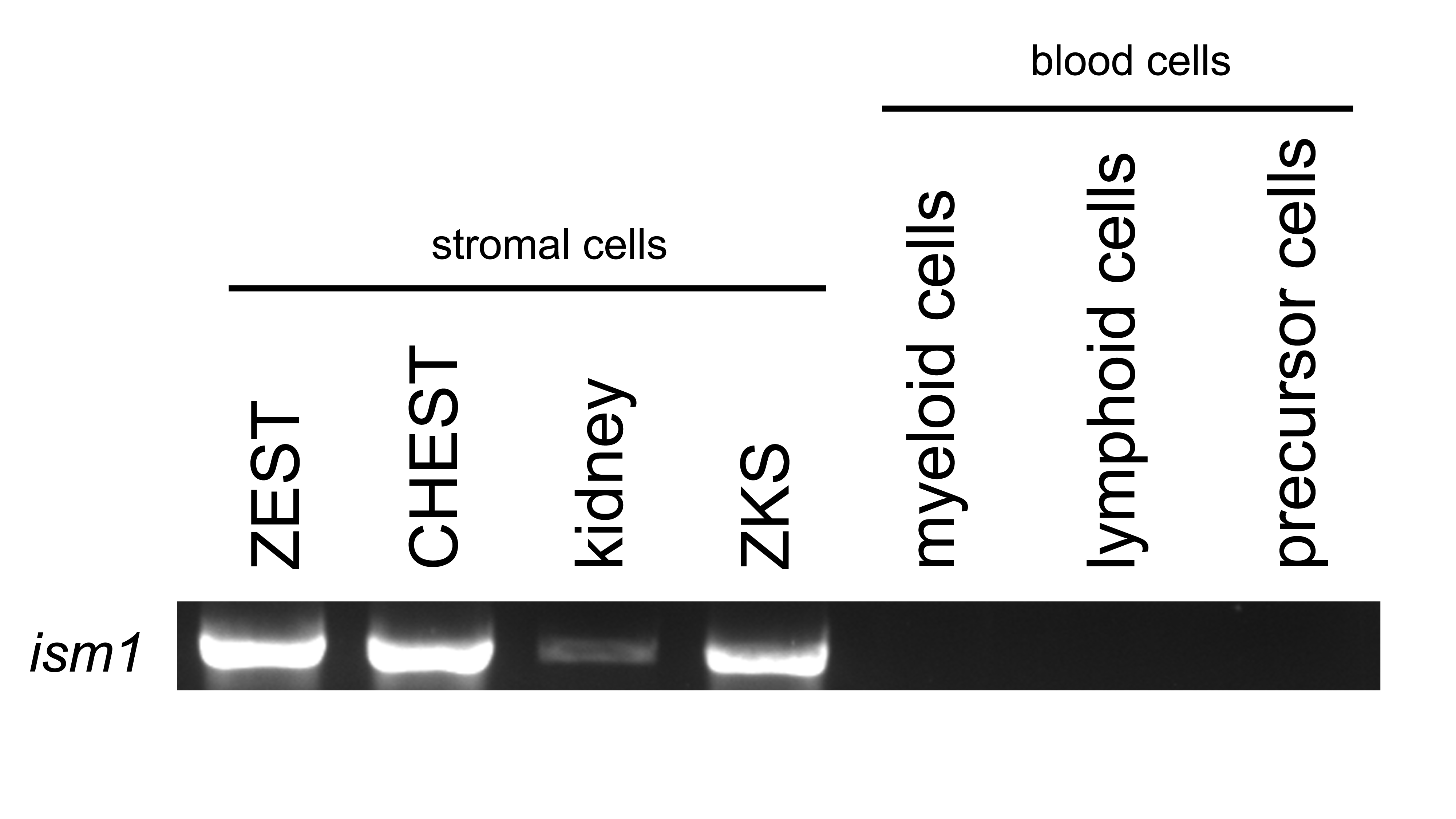

Supplement: S1 Fig — RT-PCR was performed for ism1 from ZKS, ZEST, CHEST, and kidney mRNA. Myeloid, lymphoid, and precursor cell mRNA isolated from zebrafish kidney was also interrogated for ism1 transcripts. (TIF) [file pone.0196872.s001.tif]

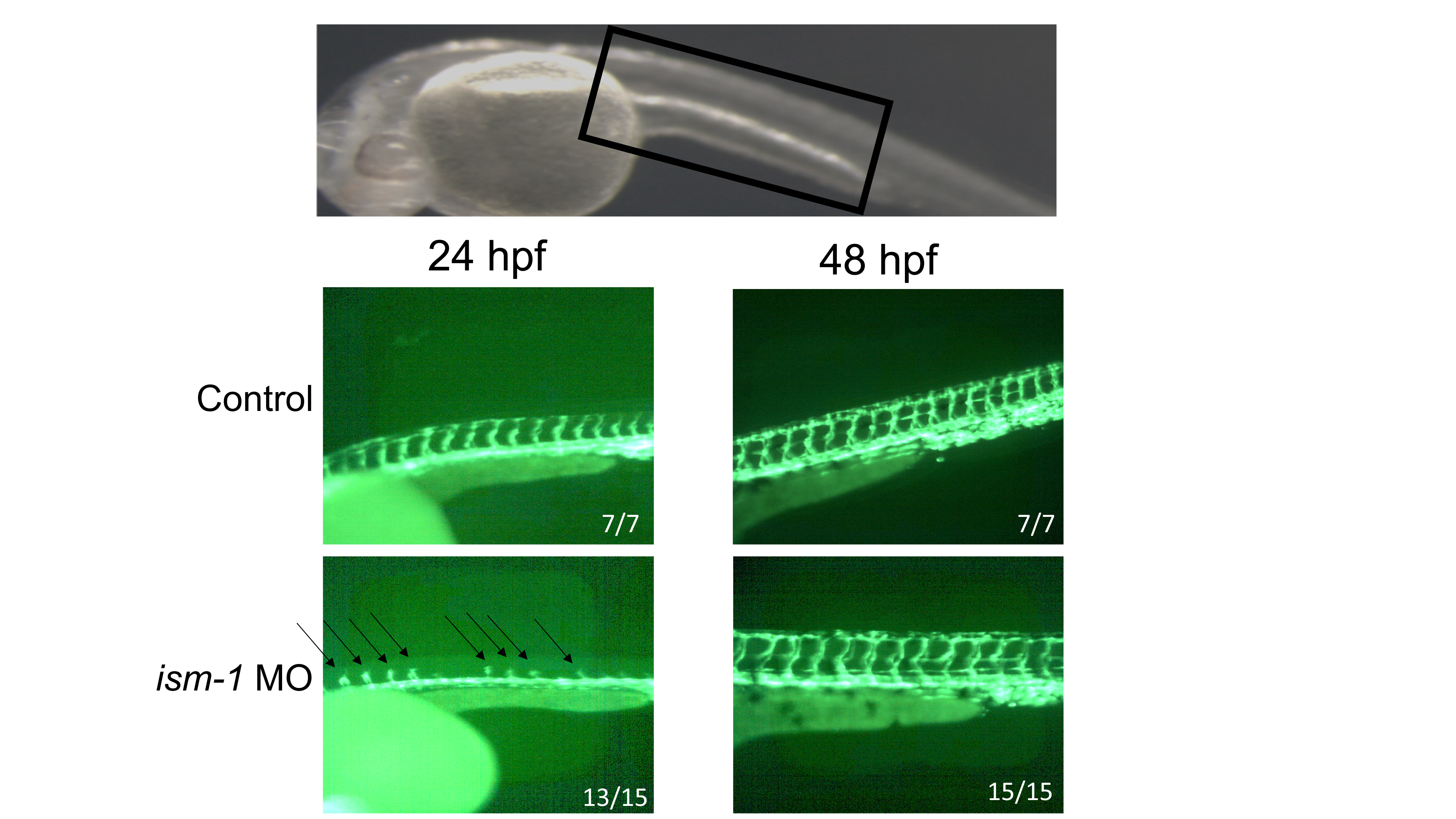

Supplement: S2 Fig — flk1:GFP single-cell-stage embryos were injected with 7 ng of ism1 MO (bottom); uninjected embryos served as controls (top). 24 hpf (left column) and 48 hpf (right column) zebrafish were visualized at 40x for flk1:GFP fluorescence within the trunk area denoted by black box in brightfield image at top center. Arrows indicate shortened intersegmental vessels in ism1 morphants. Numbers in corner of images denote the number of embryos displaying the imaged phenotype. (TIF) [file pone.0196872.s002.tif]
